# Comparison of uridine diphosphate-glycosyltransferase UGT76G1 genes from some varieties of *Stevia rebaudiana* Bertoni

**DOI:** 10.1038/s41598-019-44989-4

**Published:** 2019-06-12

**Authors:** Nader R. Abdelsalam, William A. Botros, Ahmed E. Khaled, Mohamed A. Ghonema, Shimaa G. Hussein, Hayssam M. Ali, Mohamed S. Elshikh

**Affiliations:** 1 Agricultural Botany Department, Faculty of Agriculture (Saba-Basha), Alexandria University, Alexandria, P.O. Box 21531, Bololky Egypt; 2 Department of Nucleic Acids Research, Genetic Engineering & Biotechnology Research Institute (GEBRI), City for Scientific Research and Technology Applications, Alexandria, P.O. Box 21934, Egypt; 3 Sugar Crops Research Institute, Agricultural Research Center (ARC), Ministry of Agriculture, Alexandria, P.O. Box 21616, Egypt; 4 Botany and Microbiology Department, College of Science, King Saud University, P.O. Box 2455, Riyadh, 11451 Saudi Arabia; 5 Timber Trees Research Department, Sabahia Horticulture Research Station, Horticulture Research Institute, Agriculture Research Center, Alexandria, P.O. Box 21616, Egypt

**Keywords:** Genomic analysis, Genomics

## Abstract

Stevia leaves contain various components, such as flavonoids, labdanes, chlorophylls, sterols, triterpenoids, mono-disaccharides, organic acids and inorganic salts. Stevia is known to accumulate diterpenoid steviol glycosides, which are approximately 300 times sweeter than regular sugar. Stevioside and rebaudioside A are the main diterpenic glycosides in stevia. Steviol glycosides are the secondary metabolites responsible for the sweetness of stevia. The main objectives of the present study were to determine the concentrations of diterpenic glycosides (stevioside and rebaudioside A) in three stevia varieties (*Stevia rebaudiana) via* the HPLC-UV technique and to amplify the UGT76G1 gene by PCR using gene-specific primers. The expression levels of the UGT76G1 gene were determined in the three stevia varieties. The PCR products were sequenced and analyzed, and the nucleotide sequences of the UGT76G1 gene were submitted to GenBank and assigned to the following three varieties: Egy1 (MH087463), China1 (MH087464) and Sponti (MH087465). Cluster analysis was used to separate the three varieties into two major clusters based on their phylogenetic relationship. In addition, chemical analysis was carried out to evaluate stevioside and rebaudioside A. The present study concluded that Egy1 and Sponti are closely related varieties as they fall in the same cluster, while China1 forms a separate cluster. Bioprospecting studies could be useful for selection of superior ecotypes of *Stevia rebaudiana*.

## Introduction

Stevia plants are an important source of commercial steviol glycosides (SGs)^1^. SGs are used as alternative natural sweeteners and have applications in the control of diseases caused by modern lifestyles, such as obesity, diabetes, hypertension and cardiac blockage^2–4^. Up to thirty percent of diterpenoid steviol glycosides accumulate in dry stevia leaves^5^. Steviol glycosides are sweeter than sugar and noncalorific sweeteners that are used worldwide. Eight different steviol glycosides are produced in stevia plants. Stevioside constitutes a majority of sweeteners (60~70%) with high potential medicinal value^6–8^. Rebaudioside A is of particular interest, due to the desirable flavor profile of this compound and is considered to be antidiabetic, noncariogenic and mutagenic^9^. Diterpene glycosides are currently used in different varieties of food products (pickled vegetables, dried sea food, beverages, candies, chewing gum, yogurt, *etc*.). Different techniques are used to determine the glycoside content in plants (*e.g*., gas chromatography, HPLC, LC-MS, infrared spectroscopy). HPLC is a reliable method that has been used to determine the composition of *Stevia rebaudiana*^10–13^. Plant UDP-glycosyltransferases (UGTs) are a unique group of enzymes that transfer sugar residue from an activated donor to an acceptor molecule^14–19^. UDP-glycosyltransferases were mostly unidentified until recently, and detailed functional characterization of these enzymes is only just beginning. Complete genome sequencing uncovered 112 full-length candidate UGTs in *Arabidopsis*, and these results led to the characterization of many new activities^20–24^. In *Stevia*, UDP-glycosyltransferases are involved in the production of steviol glycosides, compounds that are unique in the plant world due to their intense sweetness and high concentration in leaves^25,26^. In stevia, kaurene is transformed to steviol, the backbone of the sweet glycosides; steviol is also transformed to many glycosides by uridine-diphosphate-dependent glycosyltransferases (UGTs)^27–29^. The C-19 carboxylate and C-13 alcohol oxygenated functional groups of steviol provide attachment points for the sugar side chains that determine the identity of the glycosides. The addition of the C-13-glucose to steviol is catalyzed by UGT85C2, first yielding steviolmonoside and then steviolbioside; the addition of the C-19-glucose is catalyzed by UGT74G1, yielding stevioside^30,31^; and finally, glucosylation of the C-3′ of the glucose at the C-13 position is catalyzed by UGT76G1, yielding rebaudioside A^27,32^. The UGT76G1 gene is responsible for the conversion of stevioside to rebaudioside A and improves the organoleptic properties of steviol glycosides^33^; therefore, the present study was focused on UGT76G1. To differentiate between three stevia varieties, biochemical analysis of the three varieties was carried out, to determine the concentrations of both stevioside and rebaudioside A in stevia leaves using HPLC. Moreover, the gene expression levels of UGT76G1 in the three stevia varieties. Furthermore, the UGT76G1 gene was characterized *via* gene sequencing to determine the genetic similarity of the three stevia varieties, and the obtained sequences were submitted to GenBank to identify accession numbers.

## Material and Methods

### Sample collection

Mature leaves (3 months old) of three stevia varieties (Egy1, China1 and Sponti of *S. rebaudiana*) were collected from the middle parts of plants (between internodes 10 and 15) from the Sugar Crops Research Institute, Agricultural Research Center (SCRI-ARC), Ministry of Agriculture, Egypt. Cooperating with Botany and Microbiology Department, College of Science, King Saud University, Saudi Arabia.

### Sweet diterpene glycoside extraction

The collected leaves were oven-dried (E. Schulz & Co. Inh. Franz. Skorezewsh KOMEG Technology, China) at 50 °C and then ground to a fine powder according to^34^ with some modifications as follows: 5 g of dried leaf powder was extracted with 50 ml of hot methanol using a Soxhlet apparatus for 2 hrs. The extract was filtered using Whatman no. 1 filter paper, and the residue was re-extracted twice with methanol at room temperature. The filtrate was further concentrated in rotary flash evaporator (Type 349, James Jobling and Co. Ltd., England) at 60 °C to 10 ml, and then, 50 ml of distilled water was added to the concentrated extract. A phase separation step was performed to remove plant pigments as follows: 25 ml of diethyl ether was added to the extract in a 500-ml separatory funnel (Sigma Aldrich), and the aqueous phase was collected and extracted with butanol. Finally, the butanol upper layer was collected and refrigerated overnight at 4 °C to allow the purified glycosides to form crystals. Then, the crystals were separated by filtration and analyzed using HPLC^34^. Each stevia sample was extracted twice.

### Preparation of the stevioside standard

Stevioside standard preparation was carried out according to the method described by Nishiyama *et al*.^35^ with minor modifications as follows: dried leaves of *S. rebaudiana* Bertoni (10 g) collected from Sugar Crops Research Institute (SCRI), Agricultural Research Center (ARC), Ministry of Agriculture, Egypt, were extracted by soaking leaves in 1.0 liter of hot distilled water (85 °C) for 30 minutes. The resulting liquid fractions were filtered using a Buchner filtration system, and the leaves were then washed with an additional volume of hot water (50 ml). The aqueous solution was concentrated to 50 ml in a freeze-drier (Edwards model EF03, England). The extract was defatted by ethyl acetate followed by extraction with isobutyl alcohol (150 ml). The aqueous phase was discarded, and the organic phase was evaporated until dryness by using a rotary evaporator (Type 349, James Jobling and Co. Ltd., England) at 70 °C. The dry pellets were dissolved in hot methanol (100 ml) and allowed to crystallize overnight. The crystals were separated by filtration and dissolved in boiling methanol (50 ml) to obtain a concentrated solution. The solution was clarified with active charcoal (B.D.H. Laboratory Chemicals Division, Poole, England) and left to recrystallize. The procedure was repeated three times until the formation of colorless crystals. The pure solution of the stevioside standard was subjected to HPLC analysis.

### Analysis of SGs by HPLC

High-performance liquid chromatography (HPLC) technology can be used to directly measure the levels of steviol glycosides (rebaudioside A and stevioside) in *Stevia rebaudiana* Bertoni^36^. The levels of stevia sweetener compounds were estimated at the Central Laboratory, Faculty of Science, Alexandria University. Leaf extracts were separated and identified by HPLC according to^37^ as follows: the stevioside solution was filtered through a Millipore membrane (13 mm diameter, 0.5 μm pore size) and analyzed using HPLC with a stevioside standard as an internal standard (10 mg/ml). Different extracts of stevia leaves were injected into an HPLC instrument (Shimadzu, Tokyo, Japan; model SPD-6AV) equipped with an LC-GA UV-vis detector and an Alex C-R 4 A recorder. The separation was carried out on a Zorbax NH2 column (25 cm × 0.4 mm I.D.; Dupont, Wilmington, DE, USA) with acetonitrile (HPLC grade, Fisons Co., England) as the mobile phase (acetonitrile: water (80: 20 v/v), adjusted to pH 5 with H_3_PO_4_). The flow rate was 2 ml/min; the UV detection wavelength was 210 nm; the recorder chart speed was 20 nm/min; and the analysis was performed at ambient temperature (25 °C). Two samples per variety were analyzed, and the quantities of stevioside and rebaudioside A were calculated from the area under each peak.

### PCR amplification of the stevia UGT76G1 gene

Fresh leaf samples of three stevia varieties (three months old), namely, China1, Egy1 and Sponti, were used to extract total genomic DNA using the DNA Mini-Prep Kit (BIO BASIC, Canada). The stevia UGT76G1 gene was amplified by using gene-specific primers UGT76G1 FP (5′ AACGTCAGTCAAACCCAATG3′) and UGT76G1 RP (5′ CTCACATAACCAACAACCATCC3′) according to^33^. The PCR was performed in a 25-µl reaction mixture containing 1 µl of 100 ng/µl DNA, 1 µl of each primer at a concentration of 0.01 nmol/µl, 12.5 µl of master mix (2.5 µl of 10 × PCR buffer, 1.5 µl of 25 mM MgCl_2_, 2.0 µl of 2.5 mM dNTP, and 0.125 µl of Taq DNA polymerase) (BIOLINE, UK) and 9.5 µl of water (H_2_O). The PCR program was as follows: denaturation for 5 min at 95 °C; 35 cycles of 40 s at 94 °C, 40 s at 55 °C and 2 min at 72 °C; and 72 °C for 10 min. Then, 2% agarose gel electrophoresis with ethidium bromide was used to separate the PCR product. The image was recorded using a gel documentation system (Alpha Image, USA). Clearly separated DNA bands of PCR products (approximately 1500 bp) were cut from the gel and purified using the GF-1 AmbiClean Kit (PCR & Gel, Vivantis, WE CARE, Malaysia). Then, the purified PCR products were submitted for sequencing at Macrogene (Korea). The sequences were analyzed and compared by NCBI BLAST (http://blast.ncbi.nlm.nih.gov); the sequences were aligned to generate a phylogenetic tree by using Molecular Evolutionary Genetics Analysis (MEGA5) software. The sequences were submitted to GenBank (gb-admin@nbci.nlm.nih.gov), and each sequence was assigned a GenBank accession number.

### Gene expression analysis of UGT76G1 (qRT-PCR)

#### RNA extraction and cDNA synthesis

Total RNA was isolated from stevia leaves using the BS82314-50 Preps EZ-10 Spin Column Plant RNA Mini-Prep Kit (BIO BASIC, Canada). For cDNA synthesis, the first strand of cDNA was synthesized using M-MuLV reverse transcriptase (New England Bio Labs Inc.). The samples were incubated at 42 °C for 1 hr and then 72 °C for 10 minutes. cDNA samples were stored at −20 °C. Every 20 µl of the reverse transcription mixture contained 1 µl of template RNA, 2 µl of oligo (dT) primer, 2 µl of 10 × M-MuLV buffer, 1 µl of M-MuLV RT (200 U/µl), 1 µl of 10 mM dNTP mix, and nuclease-free water to a total volume of 20 µl.

#### Quantitative real-time PCR analysis (qRT-PCR)

Primer design for the UGT76G1 gene is recorded in Table (1). The stevia actin gene was used as an internal control for data normalization. For relative quantification of gene expression, qRT-PCR was conducted in an Eppendorf Master Cycler ep realplex using the following PCR cycling conditions: 2 min at 95 °C, followed by 40 cycles of 5 s at 95 °C, 10 s at 60 °C and 5 s at 72 °C; then, melting curve analysis was performed according to^33^. Expression of the UGT76G1 gene was determined by quantitative qRT-PCR on a Thermo Scientific PikoReal 96 real-time PCR system (www.thermoscientific.com/pikoreal) with the SYBR Green SensiFAST^TM^ SYBR^®^ No-ROX Kit (BIOLINE). Relative quantification by real-time PCR was performed in a 10 µl volume containing 1 μl of cDNA, 5 µl of 2 × SensiFAST SYPBR^®^ No-ROX mix, 0.5 µl of each primer, and 3 µl of H2O. For quantitative real-time PCR data analysis, relative expression of UGT76G1 was calculated based on the threshold cycle using the 2^−ΔΔCq^ method^38^. The expression levels of target genes were normalized using the stevia actin gene as an internal control, and the relative transcript levels were calculated as follows according to^39^.

$$\begin{array}{c}{\boldsymbol{\Delta }}\text{Cq}\,(\text{Control},\,\text{Treatment})={\bf{Cq}}({\rm{Target}}\,{\rm{gene}})-{\bf{Cq}}({\rm{Reference}}\,{\rm{gene}}),\\ \,{\rm{where}}\,{\boldsymbol{\Delta }}{\boldsymbol{\Delta }}\text{Cq}\,{\rm{expression}}={2}^{-{\rm{nCq}}39}\end{array}$$

**Table 1:** Primer set designed for qRT-PCR used in the current study.

Gene	Primer sequence	Reference
Stevia actin	F-5′ CCCGCCATGTATGTCGCCATTCAA 3′	Madhav *et al*. (2012)
	R-5′ TCAGTGAGGTCACGACCAGCAAGA 3′	
UGT76G1	F-5′ AACGTCAGTCAAACCCAATG 3′	Yang *et al*.^33^
	R-5′ CTCACATAACCAACAACCATCC 3′	

### Statistical analysis

Data analysis was performed by using the Excel software program; gene expression was examined for three biological replicates of each variety. A t-test at p < 0.05 was applied to determine significant differences in gene expression between the three varieties, and standard deviations were calculated for the means of the biological replicates.

### Compliance with Ethics requirements

This article does not contain any studies with human or animal subjects.

## Results

### Chemical analysis of stevia sweeteners

**Figure 1:**
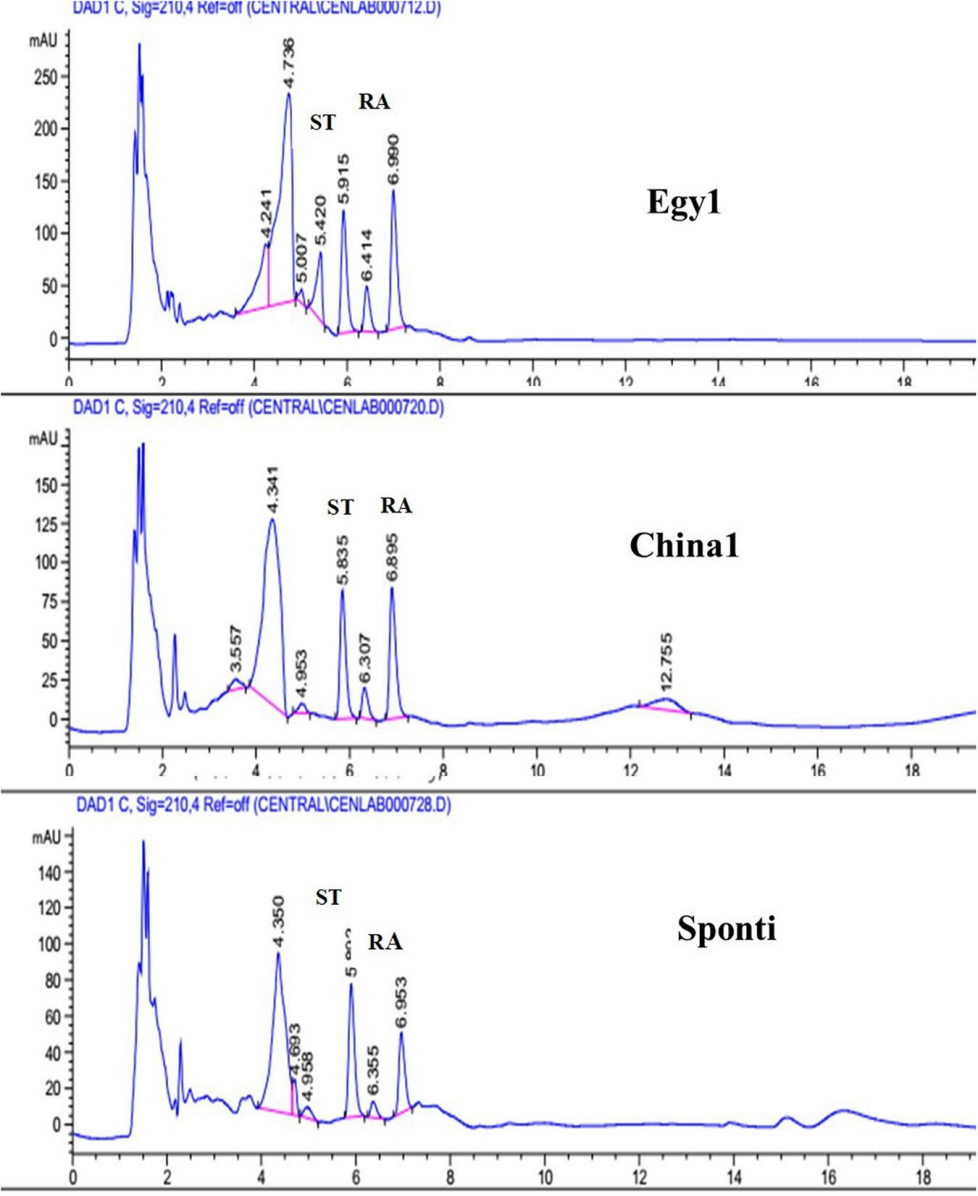
HPLC of stevia varieties Egy1 (**A**), China1 (**B**)and Sponti (**C**).

HPLC was used to determine the levels of stevioside and rebaudioside A. The results shown in Table (2) and Fig. (1A–C) indicate that the highest stevioside content was observed in the Sponti variety (21.46%), followed by China1 (0.18%) and finally Egy1 (12.27%). The range from lowest to highest value was 12.27 to 21.46% (with a 9.19% increase observed for the Sponti variety, Table 2). The highest levels of rebaudioside A were observed in China1 and Egy1 (15.54% and 14.48%, respectively), while a value of 13.02% was observed for the Sponti variety (Table 2).

**Table 2:** Percentage of the stevioside and rebaudioside A sweeteners in stevia leaves (values are the means of two readings for each variety).

Egy1		China1		Sponti	
Stevioside %	Rebaudioside A %	Stevioside %	Rebaudioside A %	Stevioside %	Rebaudioside A %
12.27	14.48	14.18	15.54	21.46	13.02

### PCR amplification of UGT76G1

**Figure 2:**
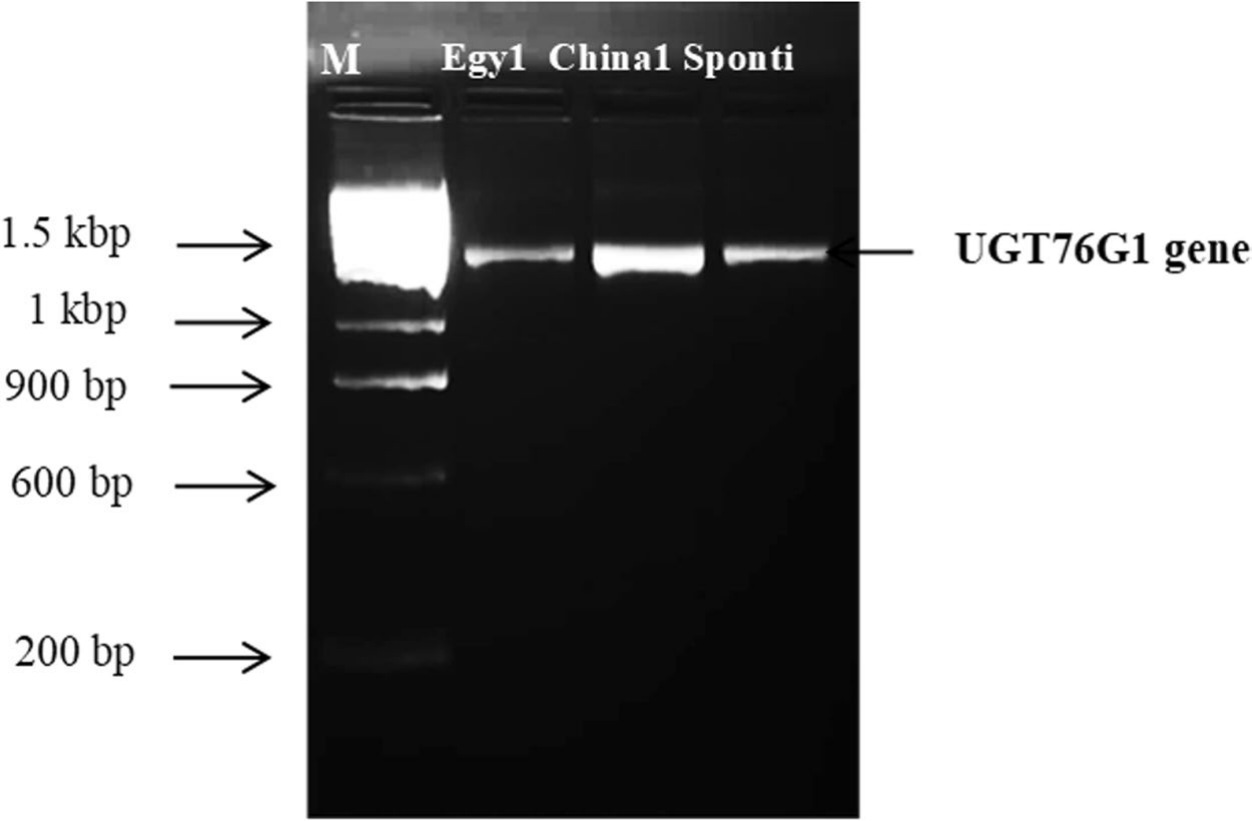
PCR products of the UGT76G1 gene amplified from Egy1, China1 and Sponti.

The UGT76G1 gene was amplified from the stevia varieties by PCR using the designed primers (UGT76G1 F and R). The PCR assay showed an amplification product of the expected size (1.51 kb), as shown in Fig. (2). These data are consistent with^33^, in which a product with same molecular weight was detected.

### UGT76G1 gene sequencing and phylogenetic analysis

**Figure 3:**
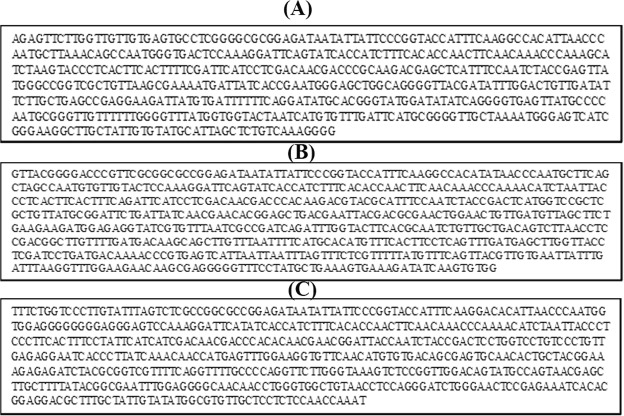
Nucleotide sequencing of the UGT76G1 gene from stevia plants of different varieties: (**A**) Egy1 (519 bp); (**B**) China1 (634); (**C**) Sponti (549).

**Figure 4:**
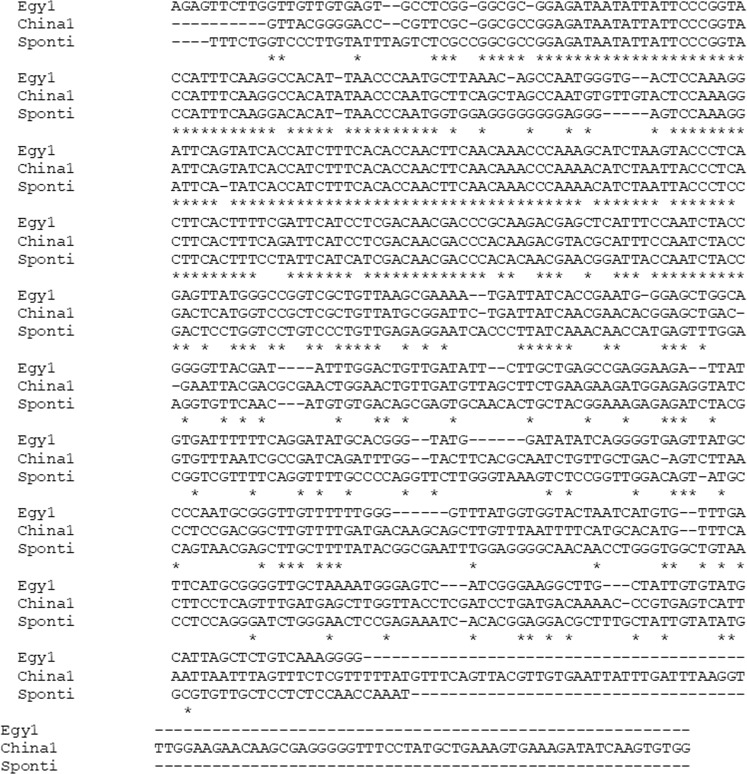
Multiple DNA sequence alignments of UGT76G1 genes with the genes from three different varieties of stevia. Completely conserved residues across all the aligned sequences are marked with an asterisk (*) below. Absent nucleotides are indicated by dashes (−).

**Figure 5:**
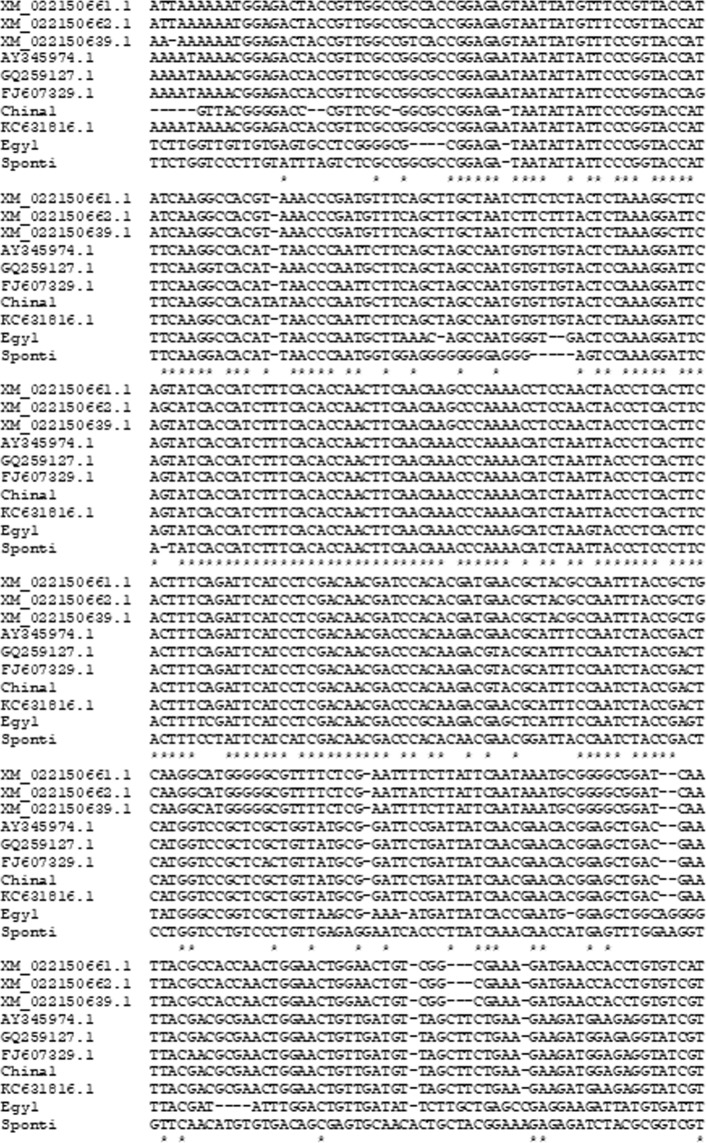
Multiple DNA sequence alignments of the UGT76G1 genes with the genes from three different varieties of stevia available GenBank. Completely conserved residues across all the aligned sequences are marked with an asterisk (*) below. Absent nucleotides are indicated by dashes (−).

**Figure 6:**
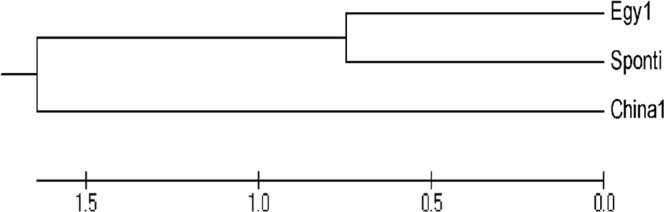
Dendrogram illustrating the phylogenetic relationship of different varieties based on DNA nucleotide sequencing of the UGT76G1 gene.

**Figure 7:**
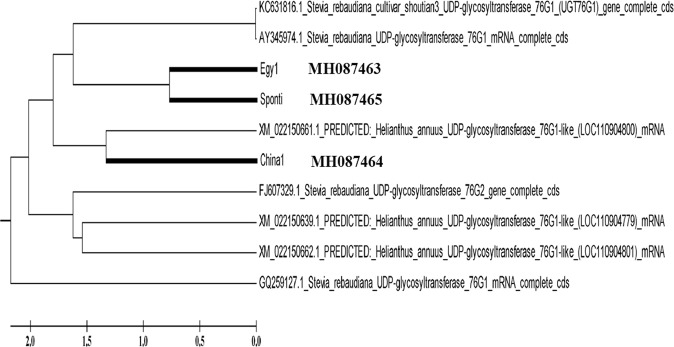
Dendrogram illustrating the phylogenetic relationship of different varieties based on DNA nucleotide sequencing of the UGT76G1 gene and comparison with the same gene listed in GenBank.

The polymerase chain reaction amplification products of the UGT76G1 gene were sequenced and analyzed to determine nucleotide similarity among the three stevia varieties, as shown in Fig. (3). Data were submitted to GenBank for identification of the accession numbers for each sequence (Table 3, Figs 4 and 5). The partial sequence of the UGT76G1 gene was aligned and compared to the sequences in GenBank. A dendrogram was generated using MEGA5 software to examine the phylogenetic relationship of UGT76G1 among the three stevia varieties, namely, Egy1, China1 and Sponti. The observed similarity might be the result of the existence of a common ancestor for Egy1 and Sponti, and this ancestor might differ from the ancestor of China1 (Fig. 6). The generated genetic similarity dendrogram for the three stevia varieties classified the populations into two major groups: Group I (Egy1 and Sponti) and Group II (China1). The results showed that Egy1 and Sponti belong to the same cluster (Figs 6 and 7).

**Table 3:** Accession numbers of the UGT76G1 gene in GenBank.

Variety	Gene	Length (bp)	Accession number
Egy1	UGT76G1	519	MH087463
China1	UGT76G1	634	MH087464
Sponti	UGT76G1	549	MH087465

### Gene expression analysis of UGT76G1

**Figure 8:**
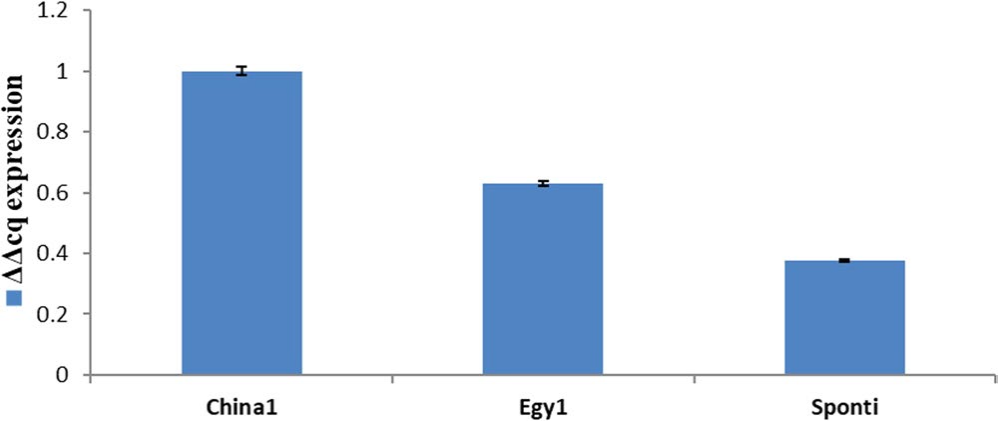
Quantitative estimation of UGT76G1 gene expression in stevia leaves.

The data presented in Table (4) and Fig. (8) indicate that the gene expression of UGT76G1 varied significantly between the three stevia varieties under investigation. Based on the obtained results, China1 showed the highest gene expression level, exhibiting a ΔΔCq value of 0.178, followed by Egy1, which exhibited a relatively low expression level (ΔΔCq value of 0.119). The lowest gene expression level was reported for the Sponti variety, which showed a ΔΔCq value of 0.074, and these values were significantly different for the three varieties (p = 0.05). The relative gene expression of UGT76G1 was significantly high between China1 and Egy1 (value of 0.002), followed by China1 and Sponti and Egy1 and Sponti (0.0001 and 0.0007, respectively). These results support the finding obtained using HPLC analysis that showed a significant increase in rebaudioside A concentrations in China1 compared to Sponti.

**Table 4:** Relative gene expression of the stevia UGT76G1 gene in three varieties using RT-quantitative PCR.

Variety	Average of 2^− ∆∆Cq^	Gene expression in stevia varieties relative to that in China1
China1	0.178 ± 0.012	1.00
Egy1	0.119 ± 0.007	0.628
Sponti	0.074 ± 0.002	0.376

## Discussion

The stevioside content observed in this study was higher than that reported by Parris *et al*.^39^, in which the concentration ranged from 2.8–5.49%^25^ and 6.98–12.16%. For rebaudioside A content, China1 exhibited the highest value (15.54%), while Sponti exhibited the lowest value (13.02%). A low value for rebaudioside content was also reported by^40^. The variation in stevioside content and rebaudioside A content was further speculated to be associated with increasing altitude, resulting in decreasing temperature and in turn in accumulation of stevioside. Recently, stevia has received much attention. Rebaudioside A represents 30–40% of total glycosides^41^, but in the current study, the rebaudioside A levels of the stevia varieties China1, Egy1 and Sponti were 15.54%, 14.48% and 13.02% of the total glycosides, respectively. Therefore, these varieties are considered good materials for the study of the synthesis of steviol glycosides. The results of the present study are consistent with those of previous studies that determined stevioside, rebaudioside A and steviol levels via different methods^42^. HPLC and NIR spectroscopy models have been used to directly measure the steviol glycoside content in *S. rebaudiana* Bertoni to decrease the cost and complexity of operation^36^. The UGT76G1 gene was amplified from the stevia varieties by PCR using the designed primers (UGT76G1 F and R). The PCR assay showed an amplification product of the expected size (1.51 kb), which is in accordance with Li *et al*.^24^. The phylogenetic analysis indicated that the three sequences were grouped with UGT76G1 in the same cluster, indicating a close relatedness between UGTSr and UGT76G1. The phylogenetic analysis showed a close relationship between UGTSr and UGT76G1^43^. The UGT76G1 obtained from the Egy1 variety (accession number MH087463) was closely related to *S. rebaudiana* KC631816.1 and AY345974.1 (UGT76G1 genes), with a nucleotide sequence similarity of 88%. Additionally, the nucleotide sequence from China1 (accession number MH087464) showed high nucleotide sequence similarity (98%) with *Stevia rebaudiana* KC631816.1, AY345974.1 and FJ607329.1 (UGT76G2 genes). In contrast, the nucleotide sequence obtained from Sponti (accession number MH087465) was closely related to KC631816.1 and AY345974.1 with a similarity of 87%. Moreover, China1 showed high similarity with XM_022150661.1 (*Helianthus annuus* UGT76G1), while Egy1, China1 and Sponti showed low similarity with FJ607329.1 (*Stevia rebaudiana* UGT76G2), XM_022150639.1 (*Helianthus annuus* UGT76G1), XM_022150662.1 (*Helianthus annuus* UGT76G1), and GQ259127.1 (*Stevia rebaudiana* UGT76G1). In a previous study, a mutation in the UGT76G1 gene was found to cause a reduction in rebaudioside A accumulation to 0.2% compared to normal plants, which usually exhibited 30–40% accumulation of total glycosides^33^. It was concluded that these three UGTs were not expressed at higher levels than any of the other UGTs. The expression of these genes was in the following order: UGT85C2 > UGT76G1 > UGT74G1. In the present study, we used qRT-PCR, a highly sensitive and specific method, to accurately measure the transcript levels of the three varieties^44^. Increased transcript levels of the UGT76G1 enzymes increased the level of the final product of the biosynthetic pathway, rebaudioside A^45,46^. Rebaudioside A accumulation is one of the most important traits contributing to the economic value of stevia crops^47^.

## Conclusion

The present study investigated the relationship among three *Stevia* varieties using HPLC and molecular techniques. Sequence analysis was used to determine the nucleotide similarity of the stevia varieties China1, Egy1, and Sponti. The three varieties were clustered into two major groups and showed close similarity to UGT76G1. Phylogenetic relationships among different sequences of *S. rebaudiana* UGT76G1 were studied. A dendrogram of genetic similarities among the three varieties was constructed. The results indicated that the three varieties were clustered into two major groups: Group I (Egy1 and Sponti) and Group II (China1). This similarity might be the result of the existence of a common ancestor for Egy1 and Sponti, and this ancestor might be different from the ancestor of China1. The results obtained for the gene expression of UGT76G1 indicated that China1 showed the highest gene expression levels compared to the other two varieties, and the gene expression level of UGT76G1 was significantly higher in China1 than in the two varieties.
